# A Machine Learning–Based Scoring System to Identify High Immunoactivity Microsatellite Stability Tumors by Quantifying Similarity to Microsatellite Instability-High Tumors in Colorectal Cancers: Development and Quantitative Study

**DOI:** 10.2196/66960

**Published:** 2025-10-16

**Authors:** Hongkai Yan, Li Jiang, Yaqi Li, Fengchong Wang, Shaobo Mo, Weiqi Sheng, Dan Huang, Junjie Peng

**Affiliations:** 1 Department of Oncology, Shanghai Medical College, Fudan University, Shanghai, China; 2 Department of Pediatric Cardiology, Xinhua Hospital Affiliated to Shanghai Jiao Tong University School of Medicine, Shanghai, China; 3 Department of Colorectal Surgery, Fudan University Shanghai Cancer Center, 270 Dong’An Road, Shanghai, 200032, China, 86 02164175590; 4 Weifang Key Laboratory of Collaborative Innovation of Intelligent Diagnosis and Treatment and Molecular Diseases, School of Basic Medical Sciences, Shandong Second Medical University, Weifang, China; 5 Weifang Ten Nanometer Biotechnology Co., Ltd., Weifang, China; 6 Department of Pathology, Fudan University Shanghai Cancer Center, Shanghai, China; 7 Institute of Pathology, Fudan University, Shanghai, China

**Keywords:** colorectal tumor, machine learning, ML, immunotherapy, immunoactivity, tumors, mutation, genetics, cancer, colorectal, colorectal cancer, microsatellite stability, immune cell, gene mutation, Cox regression, regression analysis

## Abstract

**Background:**

Microsatellite stability (MSS) colorectal cancers (CRCs) have a limited response to immune checkpoint inhibitors (ICIs) compared to microsatellite instability-high (MSI-H) CRCs. Nevertheless, previous studies have shown that some MSS CRCs are sensitive to ICIs, although established criteria for treatment justification are still lacking.

**Objective:**

This study aimed to test the tumor-infiltrating lymphocyte (TIL) features of MSS and develop a novel computational tool for the similarity prediction between MSS and MSI-H status in patients with CRC based on multiple factors.

**Methods:**

We collected and analyzed data from 188 patients with CRC, including MSI status, immune cell distributions, clinical features, and gene mutations, using statistical methods and Cox regression. An ensemble machine learning–based MSI-H score was developed using stacked extreme gradient boosting classifiers to quantify the similarity of patient data to MSI-H data based on immune cell distributions, clinical features, and gene mutations. The model was robust and could address missing input data for immune cell distributions and gene mutations.

**Results:**

The scorer performed well (mean Cohen κ of 0.40, SD 0.05, over 10 random seeds) in identifying MSI-H–like MSS samples with TIL distributions similar to genuine MSI-H CRCs. No significant difference was observed between the TIL features of MSI-H–like MSS CRCs and MSI-H CRCs. The disparity between MSI-H–like MSS CRCs and MSS CRCs potentially lies in the T regulatory cells (*P*=.09) and macrophage (*P*=.16) populations within the tumor stromal region.

**Conclusions:**

Some patients with MSS CRC presented similar immune cell distributions with high immunoactivity compared to patients with MSI-H CRC. The MSI-H score serves as a metric to quantify the similarity of MSS CRCs to MSI-H CRCs and presents a promising avenue for more personalized and effective cancer immunotherapy treatment, offering a clinical reference for potential ICI targets in MSS CRCs.

## Introduction

Colorectal cancer (CRC) is one of the leading causes of cancer death worldwide [1]. Microsatellite status divides CRCs into two subtypes: (1) deficient mismatch repair or microsatellite instability-high (MSI-H) tumors and (2) proficient mismatch repair or microsatellite stability (MSS) and microsatellite instability-low tumors [2]. These 2 subtypes are distinct in terms of clinicopathological factors, gene mutations, and the immune microenvironment [2,3]. One of the pivotal treatment modalities in the field of CRC is immunotherapy, especially the administration of immune checkpoint inhibitors (ICIs), including antiprogrammed cell death-1 (PD-1) and antiprogrammed cell death ligand 1 (PD-L1) antibodies [4]. A reliable predictor of immunotherapy response and immunoactivity is MSI-H status; notably, the Food and Drug Administration and the European Medicines Agency granted approval for the use of ICIs to treat MSI-H CRC in 2017 and 2021, respectively [5-7].

While ICIs offer an alternative to surgery and chemotherapy for MSI-H CRCs, the use of ICIs to treat MSS CRCs still lacks justification, with no guidelines for identifying high immunoactivity MSS CRCs; thus, a large population of patients with MSS CRC lacks effective treatment options [8]. However, MSS status is not an absolute marker for excluding immunotherapy. Part of MSS CRCs showed response to ICI therapies [9]. A meta-analysis provides evidence for the application of ICI therapies in nonmetastatic MSS CRCs and highlights its safety and the potential for organ preservation with this approach [10]. In addition, Motta et al [11] demonstrated that some MSS CRCs (up to 20%) harbor a similar profile, including immunological, genetic, pathological, and clinical characteristics, to MSI-H tumors. Therefore, identifying MSS CRCs with similar profiles to MSI-H CRCs could be a reasonable approach, and a strategy for achieving this is urgently needed. Tumor-infiltrating lymphocytes (TILs), a polymorphic group consisting primarily of effector T lymphocytes, regulatory T lymphocytes, natural killer cells, dendritic cells, and macrophages, are a critical feature of CRC immunology [12]. TILs are useful in immunotherapy and immunoactivity prediction [13]. Notably, the intratumoral spatial heterogeneity of TILs is an important factor for precisely stratifying prognostic immune subgroups of MSI-H CRC [14].

In this study, we developed a novel MSI-H score based on ensemble machine learning to quantify the degree of similarity of immunoactivity between patients with MSS CRC and patients with MSI-H CRC. A subgroup of patients with MSS CRC with high MSI-H scores was defined as patients with MSI-H–like MSS CRC, exhibiting MSI-H–like features in immune cell distributions, gene mutations, pathological reports, and clinical characteristics. This work paves the way for more personalized, accurate, and effective cancer immunotherapy treatments, delivering a clinical reference for identifying potential ICI targets and advancing patient care.

## Methods

### Recruitment

Data from 188 patients with stage II CRC and tissue samples were collected from the institutional database of the Fudan University Shanghai Cancer Center between 2013 and 2019. The American Joint Committee on Cancer staging system was used to determine each patient’s stage [15]. Tested by next-generation sequencing, 24 patients were classified as MSI-H. None of the patients had radiation therapy, chemotherapy, or immunotherapy before tumor resection. Clinical and pathological data were obtained from patient records and postoperative pathology reports.

### Multiplex Immunohistochemistry Staining

**Figure 1.**
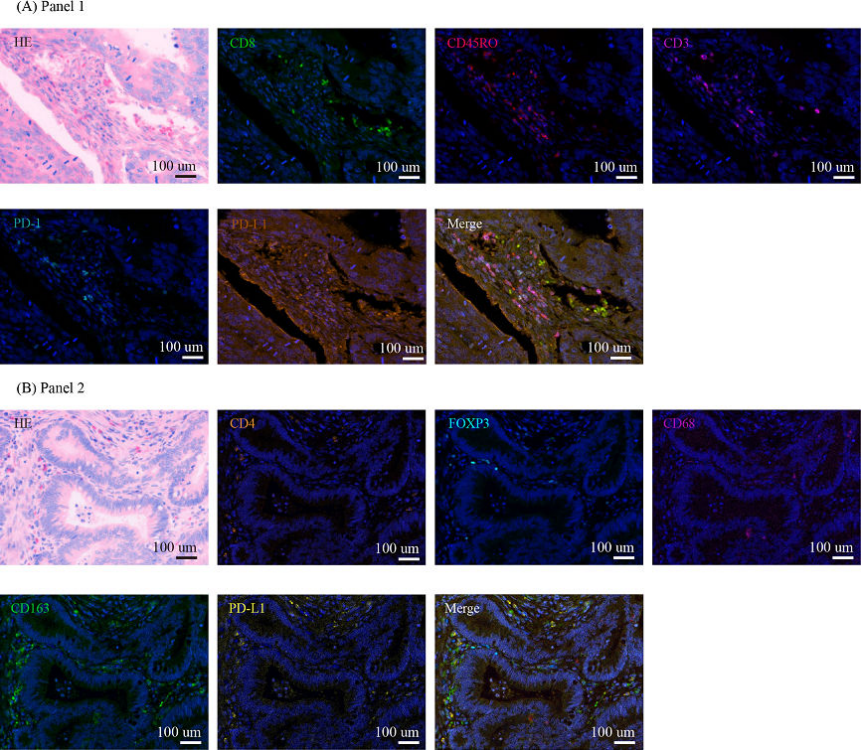
Two panels of multiplex immunohistochemistry (mIHC). (A) Representative hematoxylin and eosin (HE) and mIHC staining images of panel 1: the upper line of images represents HE staining and the staining of CD8, CD45RO, and CD3; the lower line of images represents programmed cell death-1 (PD-1) staining, programmed cell death ligand 1 (PD-L1) staining, and the merge image. (B) Representative HE and mIHC staining images of panel 2: the upper line of images represents HE staining and the staining of CD4, FOXP3, and CD68; the lower line of images represents CD163 staining, PD-L1 staining, and the merge image.

Sections (4 mm thick) were cut from formalin-fixed, paraffin-embedded CRC tissue and control tonsil tissue for multiplex immunohistochemistry (mIHC). The slides were dewaxed in xylene, rehydrated, and rinsed in graded ethanol solutions and tap water. Antibody diluent/block (72424205; PerkinElmer) was applied to block endogenous peroxidase. The slides were boiled in a Tris-EDTA buffer (pH: 9; 643901; Klinipath) and underwent microwave treatment (MWT) for antigen retrieval. Information on the primary antibodies and the corresponding fluorophores is provided in Table S1 in Multimedia Appendix 1, including 2 panels (Figure 1). One antigen required 1 round of labeling, including primary antibody incubation, secondary antibody incubation, and tyramide signal amplification (TSA) visualization, followed by labeling of the subsequent antibody. After incubation with the primary antibody for 1 hour at room temperature, the slides were incubated with Opal Polymer HRP Ms+Rb (2414515; PerkinElmer) at 37 ℃ for 10 minutes. TSA visualization was performed with the Opal 7-Color IHC Kit (NEL797B001KT; PerkinElmer) containing the fluorophores 4,6-diamidino-2-phenylindole (DAPI; Thermo Scientific) and the TSA Coumarin system (NEL703001KT; PerkinElmer). MWT was performed to remove the antibody-TSA complex with the Tris-EDTA buffer (pH: 9). TSA single-stained slides were finished with MWT, counterstained with DAPI for 5 minutes, and enclosed in Antifade mounting medium (I0052; NobleRyder).

### Image Acquisition and Analysis

Multiplexed and single-color control slides were scanned at an absolute magnification of 200× by the PerkinElmer Vectra automated multispectral microscope. Representative fields from the single-color slides were imaged, and a spectral library for unmixing was generated by inForm image analysis software (version 2.1; PerkinElmer). Index cases were stained using the multiplex method and then imaged. Channels were unmixed using the spectral library. All settings were saved within an algorithm to allow for batch analysis of multiple original multispectral images of the same tissue [16].

### Quantification of Immune Cell Densities and Classification

The nuclear morphological features were based on DAPI staining. The numbers of immune cells in each image were scored as percent cellularity (number of positive cells/number of nucleated cells). Five representative fields at 200× magnification of tissue area were selected. The densities of immune cells were segmented independently by 2 pathologists. Immune variables were classified based on the patterns of fluorochrome intensity.

### Patient Follow-Up

Patients were monitored every 3‐6 months for 3 years, then every 6‐12 months up to 5 years. Follow-ups included rectal exams, carcinoembryonic antigen (CEA) tests, annual radiological studies, and colonoscopies as needed.

### Test of MSI and CRC-Relevant Mutations

The ColonCore panel (Burning Rock) is designed for simultaneous detection of MSI status and mutations in 37 CRC-related genes (Table S2 in Multimedia Appendix 1). The MSI detection method was a read-count-distribution–based approach, using the coverage ratio of a specific set of repeat lengths as the main characteristic of each microsatellite locus. The MSI status of a sample was determined by the percentage of unstable loci in the given sample [17].

### Statistical Tests and Survival Analysis

Statistical analysis was performed and visualized by R (version 3.4.3; R Foundation for Statistical Computing), SPSS software (version 25.0; IBM Corp), and GraphPad Prism 7 software (GraphPad Software Inc). All group-wise comparisons were conducted by the 2-sided unpaired Mann-Whitney *U* test, followed by the Bonferroni procedure. The Cox proportional hazards regression model was used to assess the hazard ratios, 95% CIs, and *P* values for univariate and multivariate analysis. Variables with *P*<.10 after adjusting for common clinicopathological parameters were included in the multivariate analysis. Survival times were compared using the log-rank test. A *P* value of <.05 was considered statistically significant, and all *P* values corresponded to 2-sided statistical tests.

### Feature Engineering

The process from feature engineering to model evaluation is depicted in Multimedia Appendix 2. Categorical features were one-hot encoded as dummy variables. The mutation landscape was also one-hot encoded based on gene classes, with 2 classification stringencies, using the Database for Annotation, Visualization, and Integrated Discovery (DAVID) gene functional classification tool [18]. To further engineer the mutation landscape, we calculated the joint posterior mutation probability P(MSI|M) with the following equation:


(1) $$P\left(MSI|M\right)=\frac{P\left(MSI\right)\prod {\;}_{g_{i}}P\left(g_{i}|MSI\right)}{P\left(MSI\right)\prod {\;}_{g_{i}}P\left(g_{i}|MSI\right)+P\left(MSS\right)\prod {\;}_{g_{i}}P\left(g_{i}|MSS\right)}$$

where P(MSI|M) is the probability that a patient has MSI-high status given their mutation landscape and is based on previous probabilities and the frequencies of mutated genes in MSI-H and MSS populations, $g_{i}$ is a mutated gene in a patient’s sample, $M=\left\{g_{i}\right\}$ is a set of all detected mutated genes in the sample, $P\left(MSI\right)$ is the probability of MSI-H in a CRC sample (0.83), $P\left(MSS\right)$ is the probability of MSS in a CRC sample (0.17), $P\left(g_{i}|MSI\right)$ is the frequency of a mutated gene $g_{i}$ in a CRC MSI-H population, and $P\left(g_{i}|MSS\right)$ is the frequency of a mutated gene $g_{i}$ in a CRC MSS population. $P\left(g_{i}|MSI\right)$ and $P\left(g_{i}|MSS\right)$ were based on the findings of Serebriiskii et al [19] and 2 datasets [20,21] on cBioPortal [22,23]. Our Bayesian-based metric can explicitly incorporate previous biological knowledge, including MSI-H/MSS prevalence in populations with CRC and microsatellite status–specific mutation frequencies, and probabilistic reasoning into the modeling process. Leveraging these priors potentially enhances the model’s ability to distinguish MSI-H from MSS cases. This metric was included in the dataset along with the other features and used for model training.

Though no missing data were presented in the dataset, our model can handle missing input from users because we trained several models with varied complexities, as elaborated in the following section.

### Model Training and Deployment

Sample microsatellite status was one-hot labeled (MSI-H as 1 and MSS as 0). Multiple extreme gradient boosting (XGBoost) models [24] were trained to identify MSI-H–like tumors with different combinations of features, that is, patient metainformation, mutational landscape–derived features, and mIHC results, such as PD-L1, CD163, and CD8 mIHC staining results. The predicted likeliness of MSI-H by the models was defined as MSI-H score. Specifically, 44 models, with different combinations of features shown in Table S3 in Multimedia Appendix 1, were trained. To address potential bias caused by class imbalance, the scale_pos_weight parameter was set to the class ratio during model training.

The models were then deployed as a public web interface. As users’ data privacy is prioritized, users’ input is never stored on our server. Each model ensemble consists of 10 submodels, each trained with a distinct random seed. The final prediction for any user input is the average of the middle 6 submodel outputs, excluding the extremes. One of the XGBoost tree models in pseudocode is shown in Multimedia Appendix 3.

### Visualization and Clustering of TILs

To understand the feature importance in an unbiased and holistic way, a massive exploratory XGBoost model with all features was trained. These features include the features patient metainformation, mutational landscape–derived features, and all mIHC results. The massive model can capture the full spectrum of the MSI-H score variation and avoid the potential bias or noise introduced by the feature selection process. The feature importance was then computed using both the XGBoost built-in function and the Shapley additive explanations package [25].

Following classification by this model (threshold=0.3 defining MSI-H, chosen so that predicted MSI-H proportion approximates the epidemiologically documented prevalence of MSI-H CRC), we visualized all mIHC features of all samples by grouped box plots. For each cell type, we compared 4 groups (all MSS vs MSI-H, other MSS vs MSI-H, other MSS vs MSI-H–like MSS, and MSI-H–like MSS vs MSI-H) by 2-sided unpaired Mann-Whitney *U* test and Benjamini-Hochberg adjustment [26]. To cluster cells based on 4 comparisons, we projected each cell type into a 4D latent space using a formula measuring similarity between cell percentages of former and latter populations:


(2) $$similarity\;\left(former,latter\right)=\;\left\{ \begin{array}{ll} p_{adj}\frac{median\left(r_{former}\right)-median\left(r_{latter}\right)}{\sqrt{{\left(median\left(r_{former}\right)-median\left(r_{latter}\right)\right)}^{2}}},\;\; & \;if\;median\left(r_{former}\right)\neq median\left(r_{latter}\right)\\ p_{adj}\;,\;\; & otherwise \end{array}\right.$$

where *r*_former_ and *r*_latter_ represent the percentages of a specific cell type (eg, CD8+ cells) measured in individual samples belonging to the “former” and “latter” groups, respectively. *p_adj_* is the adjusted *P* value of a comparison. We then performed hierarchical clustering based on Euclidean distance in latent space with complete linkage [27].

### Feature and Model Evaluation

Model generalizability was assessed by training models with identical hyperparameters through stratified 5-fold cross-validation. Cohen κ coefficients were computed on each hold-out fold, and the mean Cohen κ was computed based on the 5 κ’s, with greater κ’s indicating better model performance. This training and validation process was repeated 10 times with different random stratified splits and model initializations.

### Ethical Considerations

Ethics approval was obtained from the Ethics Committee of Fudan University Shanghai Cancer Center, and informed consent was obtained from all participants (1808190‐12). Neither the patients nor the public were involved in this study (ie, only database tissue samples and data from patient records and postoperative pathology reports were used). All patient data collected for this study were deidentified prior to analysis.

## Results

### Higher TIL Infiltration in the Stromal Region Than in the Tumor Region

**Figure 2.**
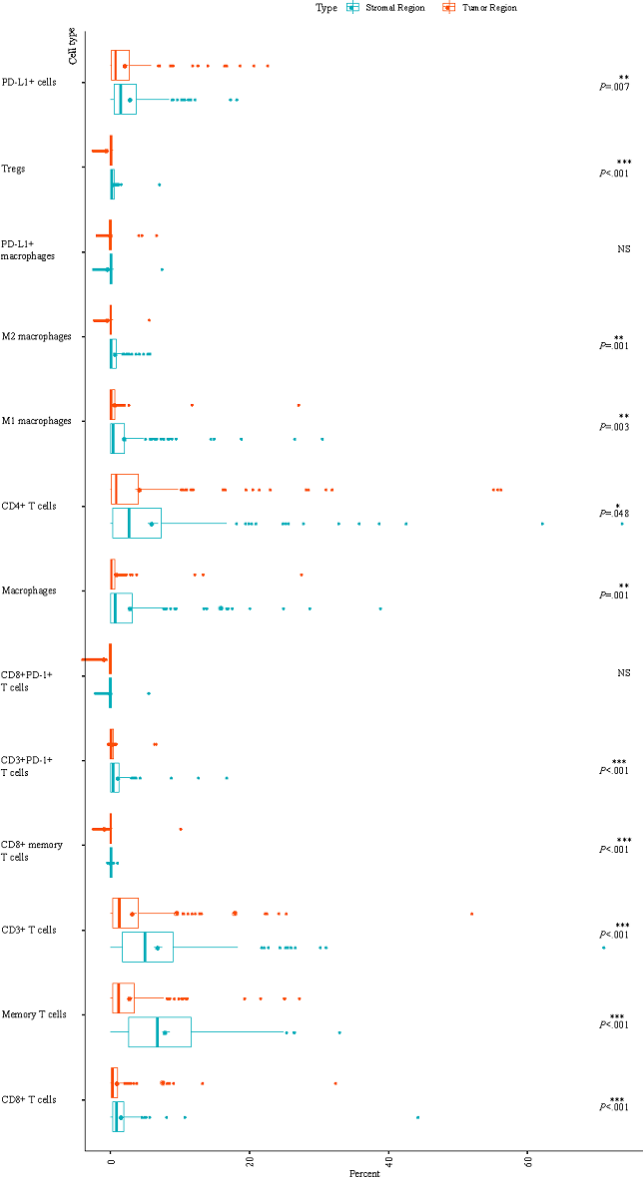
The difference in tumor-infiltrating lymphocytes between the stromal region and tumor region. Each cell type ratio of relevant samples is shown by a box plot. The cell percentage difference in the stromal region and tumor region was compared, and all *P* values were adjusted with the Bonferroni procedure and are shown on the right side. NS: not significant; PD-1: programmed cell death-1; PD-L1: programmed cell death ligand 1; Treg: regulatory T cell. *P<.05; **P<.01; ***P<.001.

TILs were analyzed using the mIHC method. Significant differences were found between the stromal region and the tumor region (Figure 2). The stromal region showed a higher prevalence of CD3+ T cells (*P*<.001), CD8+ T cells (*P*<.001), memory T cells (*P*<.001), CD8+ memory T cells (*P*<.001), CD3+ PD-1+ T cells (*P*<.001), CD4+ T cells (*P*=.048), regulatory T cells (Tregs; *P*<.001), macrophages (*P*=.001), M1 macrophages (*P*=.003), M2 macrophages (*P*=.001), and PD-L1+ cells (*P*=.007) than the tumor region. However, no significant difference was observed for CD8+ PD-1+ T cells and PD-L1+ macrophages between the stromal region and tumor region.

### Prognostic Impact of Clinical Characteristics, MSI Status, and Immune Cell Infiltration

Variables (Table 1) commonly collected in clinics and related to prognosis or with a *P* value <.10 in univariate analysis (Tables S4 and S5 in Multimedia Appendix 1) were analyzed using the Cox proportional hazards regression model (Table S6 in Multimedia Appendix 1). Microsatellite status was not linked to overall survival or disease-free survival in multivariate analysis. Significant overall survival predictors included age, CEA, M1 macrophage (CD68+ CD163–) infiltration in stromal region, and PD-1+ T cell (CD3+ PD-1+) infiltration in tumor region. Significant disease-free survival predictors included age, CEA, tumor differentiation, and CD8+ T cell (CD8+) infiltration in tumor region (Table S6 in Multimedia Appendix 1).

**Table 1.** Clinical characteristics related to microsatellite instability-high (MSI-H) and microsatellite stability (MSS) status.

Characteristic		Patients, n	MSS tumor (n=164), n (%)	MSI-H tumor (n=24), n (%)	*P* value
Sex					.82
	Male	106	93 (57)	13 (54)	
	Female	82	71 (43)	11 (46)	
Age (y)					.71
	<65	111	96 (59)	15 (63)	
	≥65	77	68 (41)	9 (38)	
Mucinous					.006
	No	152	138 (84)	14 (58)	
	Yes	36	26 (16)	10 (42)	
Differentiation					.002
	Poor	40	29 (18)	11 (50)	
	Moderate to well	140	129 (82)	11 (50)	
T stage					.59
	T3	88	78 (48)	10 (42)	
	T4	100	86 (52)	14 (58)	
Tumor site					<.001
	Right	52	36 (22)	16 (67)	
	Left	52	49 (30)	3 (13)	
	Rectum	83	78 (48)	5 (21)	
Lymphovascular invasion					.43
	No	149	128 (78)	21 (88)	
	Yes	39	36 (22)	3 (13)	
Perineural invasion					.86
	No	136	119 (73)	17 (71)	
	Yes	52	45 (27)	7 (29)	
CEA^a^ (ng/ml)					.94
	<5	124	108 (66)	16 (67)	
	≥5	64	56 (34)	8 (33)	
Chemotherapy					.45
	No	76	68 (41)	8 (33)	
	Yes	112	96 (59)	16 (67)	
Radiotherapy					>.99
	No	163	142 (92)	21 (91)	
	Yes	15	13 (8)	2 (9)	

a CEA: carcinoembryonic antigen.

### TIL Distribution in MSI-H CRCs, All MSS CRCs, MSI-H–Like MSS CRCs, and Other MSS CRCs

**Figure 3.**
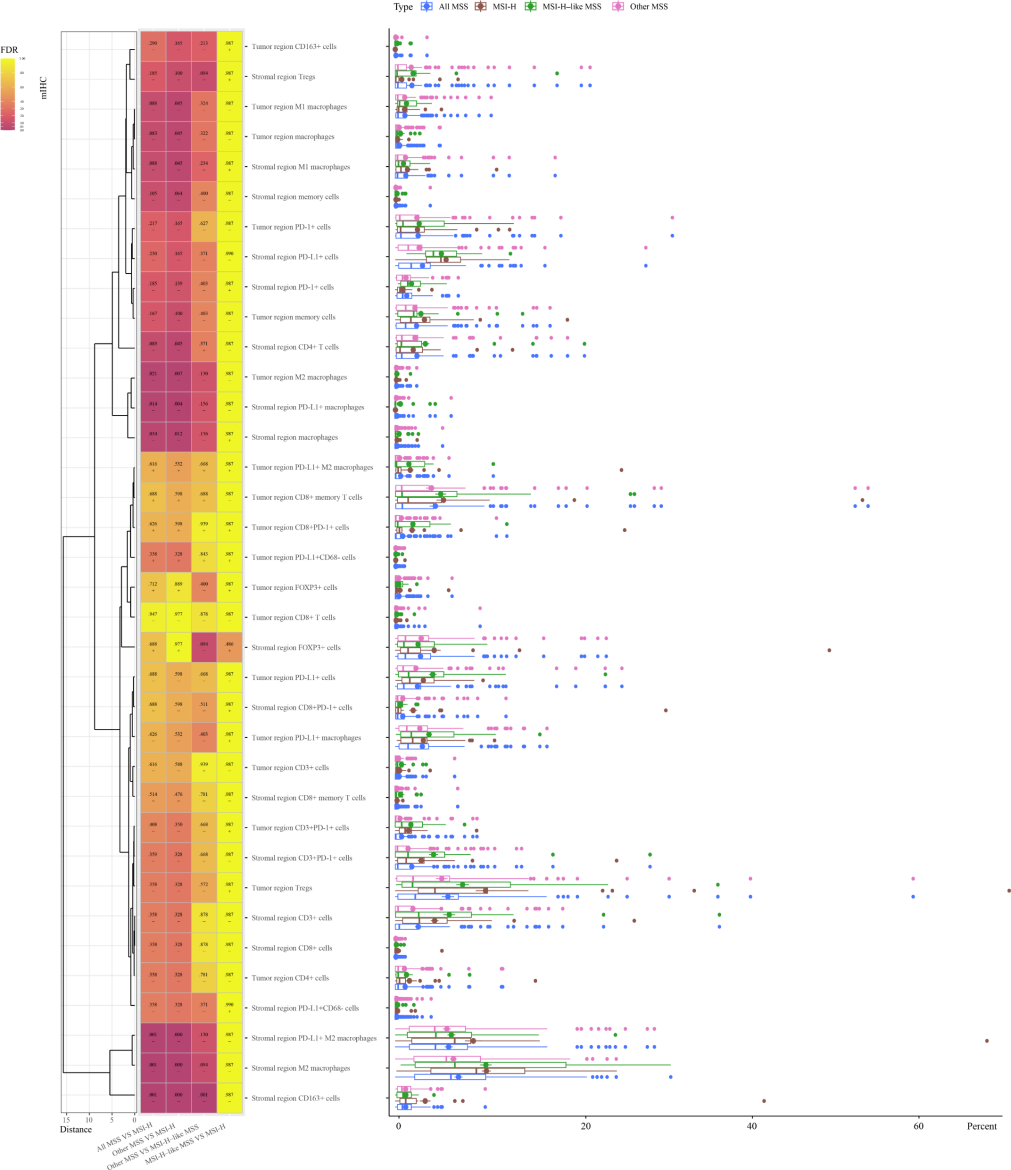
Tumor-infiltrating lymphocyte (TIL) distributions in microsatellite instability-high (MSI-H), all microsatellite stability (MSS), MSI-H–like MSS, and other MSS colorectal cancers (CRCs). The box plot on the right displays the percentages of a cell type in relevant samples for each class. Cell percentage differences were compared, and *P* values were adjusted and are presented in the heat map (*P*<.001 when adjusted *P* values were <.001 due to rounding), along with the median comparison result between the 2 populations (+: former population median>latter population median; -: former population median<latter population median). FDR: false discovery rate; mIHC: multiplex immunohistochemistry; PD-1: antiprogrammed cell death-1; PD-L1: antiprogrammed cell death ligand 1; Treg: regulatory T cell.

The mIHC experiment was performed to examine the TILs in all CRCs (Figure 3). MSI-H CRCs exhibited significantly higher infiltration of TILs compared to MSS CRCs in both the tumor region and stromal region. Specifically, MSI-H CRCs had a more abundant presence of PD-L1+ M2 macrophages (*P*=.001), CD163+ cells (*P*=.001), PD-L1+ macrophages (*P*=.01), M2 macrophages (*P*=.001), and macrophages (*P*=.03) in the stromal region, as well as M2 macrophages in the tumor region (*P*=.02).

MSI-H–like MSS CRCs exhibited TIL infiltration patterns akin to MSI-H CRCs but distinct from other MSS CRCs. No significant difference was observed between MSI-H–like MSS and genuine MSI-H CRCs (the fourth column in Figure 3). Compared to other MSS CRCs, MSI-H–like MSS CRCs showed higher infiltration of CD163+ cells in the stromal region (*P*=.001; the box plot and the third column in Figure 3) and potentially increased levels of PD-L1+ M2 macrophages (*P*=.13), FOXP3+ cells (*P*=.09), Tregs (*P*=.09), PD-L1+ macrophages (*P*=.16), M2 macrophages (*P*=.09), and macrophages (*P*=.16) in the stromal region, as well as M2 macrophages (*P*=.13) in the tumor region. The distinct infiltration patterns of TILs indicate that heightened presence of macrophages and Tregs are key factors in distinguishing MSI-H–like MSS CRCs from MSS CRCs.

Macrophages were also found to be significantly more abundant in genuine MSI-H CRCs than in other MSS CRCs (the second column in Figure 3). Specifically, in the stromal region, PD-L1+ M2 macrophages (*P*<.001), CD163+ cells (*P*<.001), PD-L1+ macrophages (*P*=.004), M2 macrophages (*P*<.001), M1 macrophages (*P*=.045), CD4+ T cells (*P*=.045), and macrophages (*P*=.01) were significantly more abundant in MSI-H CRCs than in other MSS CRCs. In the tumor region, M2 macrophages (*P*=.007), M1 macrophages (*P*=.045), and macrophages (*P*=.045) were found to be significantly increased in MSI-H CRCs compared with other MSS CRCs.

The TIL distribution shows that the model performed well. The scorer, which was trained and validated on only 3 types of lymphocytes, classified MSI-H–like MSS CRC samples with similar TIL distributions as MSI-H CRC samples (the fourth column in Figure 3) rather than MSS CRC samples (the third column in Figure 3), despite most features of TILs (15 other lymphocytes) being unknown to the model. In addition, as anticipated, the heat map in Figure 3 (second and third columns) revealed that other MSS CRC samples exhibited a slightly closer TIL distribution to MSI-H–like MSS CRC samples than to MSI-H CRC samples.

The feature importance in a large predictive model is described in Multimedia Appendix 4. TIL features from the whole or stromal region were more predictive than the tumor region alone for the MSI-H status. Top features for MSI-H score predictor included macrophage subtypes, mutational landscape, and immune cell distributions.

### MSI-H Score Predictor Generalization Ability Affected by Feature Number and Type

**Figure 4.**
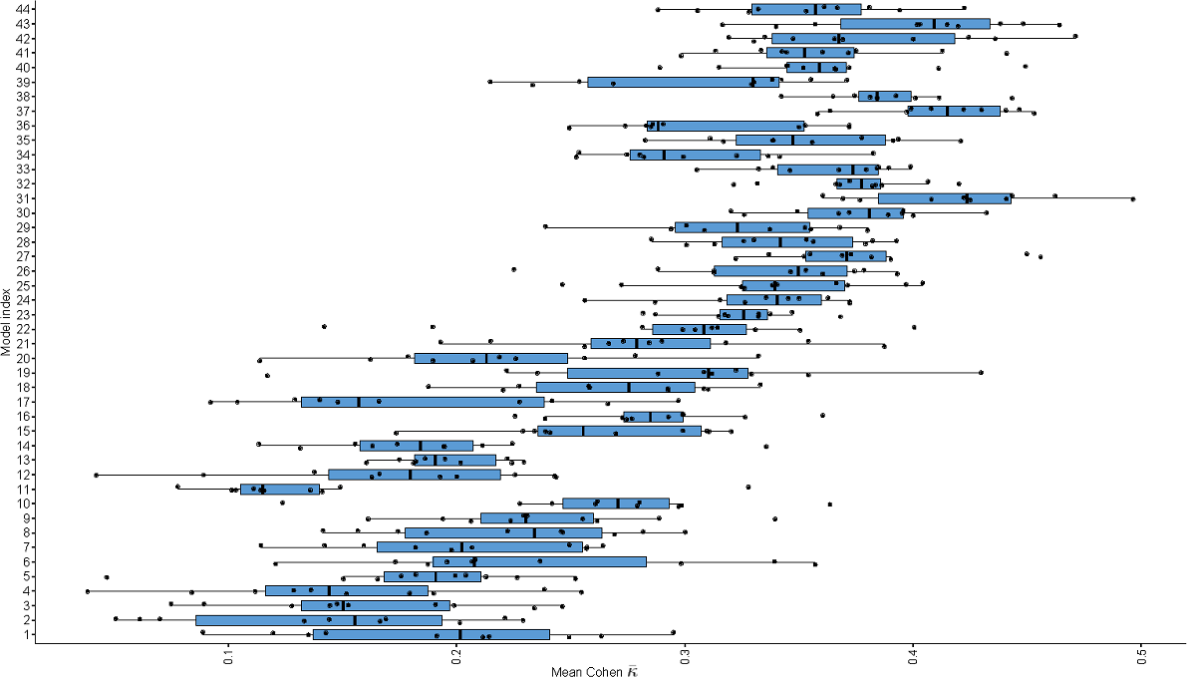
Box plot of mean Cohen κ values evaluated through 5-fold cross-validation repeated over 10 random seeds for each model. In general, as model complexity increases, the model’s ability to generalize tends to improve, as indicated by higher Cohen *κ* values.

**Figure 5.**
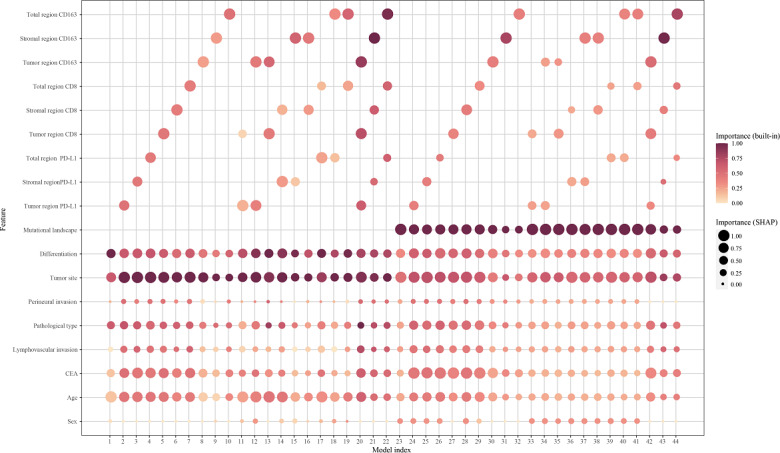
Bubble chart of feature importance. Each bubble represents a feature (y-axis) and its importance in the model (x-axis), as computed by using the native “gain” importance metric (built-in) from extreme gradient boosting (XGBoost; a darker bubble color indicates higher importance) and Shapley additive explanations (SHAP; a larger bubble area indicates higher importance). Mutation landscapes and tumor sites consistently have relatively dark, large bubbles, indicating their importance in the model. CEA: carcinoembryonic antigen.

Increasing the number of features generally enhanced $\bar{\kappa }$, indicating better generalization performance (Figure 4). However, models incorporating PD-L1 mIHC staining tended to exhibit lower $\bar{\kappa }$ compared to those without, likely due to noise in PD-L1 measurements, as evidenced by the high SD of $\bar{\kappa }$ for models 2 to 4. This noise effect was mitigated by increasing model complexity; for example, model 44 had a greater $\bar{\kappa }$ than model 41, despite including PD-L1. Feature importance analysis (Figure 5) revealed that while PD-L1 remained relevant, its importance diminished as models became more complex, suggesting that sophisticated models learned to filter out noise and extract useful information from PD-L1. The variable Spearman correlation matrix heat map is shown in Multimedia Appendix 5. In addition, an MSI-H scorer web interface is freely accessible [28].

## Discussion

### Principal Findings

Immunotherapy has been successful for treating MSI-H CRCs but is not as effective in MSS CRCs, which comprise the majority of CRCs. Thus, we developed a machine learning–based MSI-H predictor to generate a robust and reliable score that can capture the complexity and heterogeneity of CRC and better target patients with MSS CRC who may benefit from immunotherapy. Our study also provides insights into the immune landscape of CRC and the role of immune cell distributions, clinical features, and gene mutations in influencing MSI status. This CRC prognostic study mostly agrees with our previous research [29] and with findings from other authors [30]. For example, according to our results, TIL infiltration, primarily by macrophages or CD163+ cells, was significantly higher in MSI-H CRCs than in MSS CRCs (Figure 3), consistent with previous studies [31].

We observed a higher abundance of TIL subsets in the stromal region than in the tumor region, indicating a more active immune response in the stromal region (Figure 2). Comparing the MSI predictive performance of models 42, 43, and 44 (Figure 4) also highlights the importance of stromal TILs. Regional disparities underscore the importance of analyzing the complete tumor region for comprehensive insights. Our scorer successfully identified MSI-H–like MSS samples with TIL distributions similar to genuine MSI-H CRCs (Figure 3). In addition, the balance of proinflammatory and anti-inflammatory scale is an important feature for immunological characters. Macrophages can be classified into 2 main subtypes: M1 macrophages with proinflammatory and antitumor functions and M2 macrophages with anti-inflammatory and protumor functions. The ratio of M1/M2 macrophages may influence immunotherapy outcomes, reflecting the balance between proinflammatory and anti-inflammatory signals in the tumor microenvironment [32]. Tregs are frequently known to be immunosuppressive and can predict both the host immune response and chemotherapeutic response [33]. Both macrophages and Tregs are important in the regulation of immunoactivity. As is shown in our results, the distribution of macrophages and Tregs appears to be important in differentiating MSI-H–like MSS CRCs from other MSS CRCs based on TIL infiltration patterns (Figure 3). By comparing $\bar{\kappa }$ variations within model sets and between specific model set pairs (Figure 4)—including model 5/6/7 versus 13/16/19, 27/28/29 versus 35/38/41, 2/3/4 versus 12/15/18, 24/25/26 versus 34/27/40, 11/14/17 versus 20/21/22, and 33/36/39 versus 42/43/44—we observed that CD163 mIHC result increased the predictive value for whole-tumor MSI scores but reduced it for tumor region scores. To better understand the differences in M2 macrophages and Tregs between MSS CRCs and MSI-H CRCs, further research on their function in CRCs is necessary.

Our analysis revealed that PD-L1+ M2 macrophages in the total region, mutational landscape, CD163+ cells in the stromal region, PD-L1+ M2 macrophages in the stromal region, and tumor site were the most important features for predicting MSI-H status (Multimedia Appendix 4), aligning with other research results [2,3]. Macrophages can express PD-L1 and interact with PD-1+ T cells, which may affect the response to immunotherapy [34]. However, PD-L1+ macrophages potentially indicate M1-like polarization profiles [35]. The stroma is important because it can influence the extracellular matrix formation, angiogenesis, immune response, and therapeutic resistance of tumors [3]. The importance of the mutational landscape for prediction is widely known [36]. We did not find an obvious immunological explanation for why tumor site would impact the similarity between MSI-H and MSS. Further study is needed to clarify the underlying mechanisms. Moreover, we observed that feature number and type influenced the generalization ability of the MSI-H score prediction models (Figure 4 and Figure 5). This suggests that the omission of diverse variables requires specific computational models, and our machine learning scorer is adept at incorporating all such considerations, thereby highlighting our advantage.

On the basis of our results, we proposed a hypothesis regarding the changes that occur in MSI-H–like MSS CRCs compared to other MSS CRCs. MSI-H–like MSS CRCs foster an immunosuppressive microenvironment with M2 macrophages, Tregs, and PD-L1 that inhibits T cell responses [37]. However, there are enough T cells present that can be reactivated upon PD-1/PD-L1 blockade, leading to the sensitivity of MSI-H–like MSS CRCs to ICIs. The abundance of macrophages suggests that there may be some M1-like populations that, when disinhibited, promote antitumor immunity. Detailed differences in immune cell populations and their functions in MSI-H–like MSS CRCs and other MSS CRCs should be further investigated to understand the mechanisms underlying the differential response to immunotherapy. Furthermore, future clinical trials could be conducted to evaluate ICI treatment between patients with MSI-H–like MSS CRC and other patients with MSS CRC with low MSI-H scores.

### Limitations

Limitations of our study include the lack of internal or external validation of the MSI-H score in patients with MSS CRC receiving immunotherapy and the absence of investigation into the underlying molecular mechanisms. Further research and clinical trials are needed to validate our MSI-H score and elucidate the associated mechanisms.

### Conclusions

In conclusion, our study revealed significant variations in TIL distribution across tumor regions and MSI status. Integrating clinical, TIL, and mutational data, we developed a robust MSI-H scorer that captures CRC’s complexity and heterogeneity. Macrophages, gene mutations, and tumor site emerged as key predictors. MSI-H–like MSS CRCs exhibited TIL infiltration patterns with high immunoactivity similar to MSI-H CRCs, distinctly different from other MSS CRCs. Our privacy-protected MSI-H score predictor is freely available on the web, enabling clinical and research applications.

## Supplementary material

10.2196/66960 Multimedia Appendix 1 Detailed information on the experimental protocols, genetic panels, model specifications, and statistical analyses performed in this study, as provided by 6 supplementary tables.

10.2196/66960 Multimedia Appendix 2 The flowchart of feature engineering, model training, deployment, and feature and model evaluation.

10.2196/66960 Multimedia Appendix 3 An example of one of the extreme gradient boosting tree models in pseudocode.

10.2196/66960 Multimedia Appendix 4 Feature importance in a large predictive model.

10.2196/66960 Multimedia Appendix 5 Heat map of Spearman correlation coefficients between all pairs of variables (features and targets).

## Abbreviations

CEA: carcinoembryonic antigen
CRC: colorectal cancer
DAPI: 4,6-diamidino-2-phenylindole
DAVID: Database for Annotation, Visualization, and Integrated Discovery
ICI: immune checkpoint inhibitor
mIHC: multiplex immunohistochemistry
MSI-H: microsatellite instability-high
MSS: microsatellite stability
MWT: microwave treatment
PD-1: antiprogrammed cell death-1
PD-L1: antiprogrammed cell death ligand 1
TIL: tumor-infiltrating lymphocyte
Treg: regulatory T cell
TSA: tyramide signal amplification
XGBoost: extreme gradient boosting
