# Supplementary material for: A Machine Learning–Based Scoring System to Identify High Immunoactivity Microsatellite Stability Tumors by Quantifying Similarity to Microsatellite Instability-High Tumors in Colorectal Cancers: Development and Quantitative Study
Source: JMIR Form Res. 2025 Oct 16;9:e66960. doi: 10.2196/66960 (PMC12530644; doi:10.2196/66960)
Supplement: Multimedia Appendix 1 [file formative-v9-e66960-s001.doc]

**Supplementary Table** 1. Immunohistochemical multiplex staining protocol

| **Primary antibody** | **Provider** | **Clone** | **Catalogue number** | **Concentration** |
| --- | --- | --- | --- | --- |
| **panel1** |  |  |  |  |
| CD3 | Zsbio | UMAB54 | ZM-0417 | 1:50 |
| CD8 | Zsbio | EP334 | ZA-0508 | 1:100 |
| CD45RO | Zsbio | UCH-L1 | ZM-0055 | 1:200 |
| PD-1 | Zsbio | UMAB199 | ZM-0381 | 1:100 |
| PD-L1 | Roche | SP142 | 740-4859 | 1:25 |
| **panel2** |  |  |  |  |
| CD4 | Zsbio | UMAB64 | ZM-0418 | 1:1 |
| FoxP3 | Abcam | 236A/E7 | Ab20034 | 1:400 |
| PD-L1 | Roche | SP142 | 740-4859 | 1:25 |
| CD68 | Zsbio | KP1 | ZM-0060 | 1:500 |
| CD163 | Zsbio | 10D6 | ZM-0428 | 1:100 |

**Supplementary Table 2. 37 colorectal cancer (CRC)**-related genes in ColonCore panel

| *TP53* | *BRCA2* |
| --- | --- |
| *KRAS* | *BMPR1A* |
| *MSH6* | *STK11* |
| *MUTYH* | *MLH1* |
| *EPCAM* | *PTCH1* |
| *MET* | *EGFR* |
| *ERBB2* | *SMAD4* |
| *PIK3CA* | *NRAS* |
| *BRAF* | *PDGFRA* |
| *PTEN* | *SDHD* |
| *APC* | *BLM* |
| *POLE* | *GREM1* |
| *POLD1* | *AKT1* |
| *BRCA1* | *HRAS* |
| *GALNT12* | *MSH2* |
| *ATM* | *CHEK2* |
| *KIT* | *PMS2* |
| *CDH1* | *SDHB* |
| *PMS1* |  |

**Supplementary Table 3**. Model configurations

| Model index | Patient meta information | mutational landscape-derived features | PD-L1 mIHC result in tumor regions | PD-L1 mIHC result in stromal regions | PD-L1 mIHC result in total region | CD8 mIHC result in tumor regions | CD8 mIHC result in stromal regions | CD8 mIHC result in total region | CD163 mIHC result in tumor regions | CD163 mIHC result in stromal regions | CD163 mIHC result in total region |
| --- | --- | --- | --- | --- | --- | --- | --- | --- | --- | --- | --- |
| 1 | with | without | without | without | without | without | without | without | without | without | without |
| 2 | with | without | with | without | without | without | without | without | without | without | without |
| 3 | with | without | without | with | without | without | without | without | without | without | without |
| 4 | with | without | without | without | with | without | without | without | without | without | without |
| 5 | with | without | without | without | without | with | without | without | without | without | without |
| 6 | with | without | without | without | without | without | with | without | without | without | without |
| 7 | with | without | without | without | without | without | without | with | without | without | without |
| 8 | with | without | without | without | without | without | without | without | with | without | without |
| 9 | with | without | without | without | without | without | without | without | without | with | without |
| 10 | with | without | without | without | without | without | without | without | without | without | with |
| 11 | with | without | with | without | without | with | without | without | without | without | without |
| 12 | with | without | with | without | without | without | without | without | with | without | without |
| 13 | with | without | without | without | without | with | without | without | with | without | without |
| 14 | with | without | without | with | without | without | with | without | without | without | without |
| 15 | with | without | without | with | without | without | without | without | without | with | without |
| 16 | with | without | without | without | without | without | with | without | without | with | without |
| 17 | with | without | without | without | with | without | without | with | without | without | without |
| 18 | with | without | without | without | with | without | without | without | without | without | with |
| 19 | with | without | without | without | without | without | without | with | without | without | with |
| 20 | with | without | with | without | without | with | without | without | with | without | without |
| 21 | with | without | without | with | without | without | with | without | without | with | without |
| 22 | with | without | without | without | with | without | without | with | without | without | with |
| 23 | with | with | without | without | without | without | without | without | without | without | without |
| 24 | with | with | with | without | without | without | without | without | without | without | without |
| 25 | with | with | without | with | without | without | without | without | without | without | without |
| 26 | with | with | without | without | with | without | without | without | without | without | without |
| 27 | with | with | without | without | without | with | without | without | without | without | without |
| 28 | with | with | without | without | without | without | with | without | without | without | without |
| 29 | with | with | without | without | without | without | without | with | without | without | without |
| 30 | with | with | without | without | without | without | without | without | with | without | without |
| 31 | with | with | without | without | without | without | without | without | without | with | without |
| 32 | with | with | without | without | without | without | without | without | without | without | with |
| 33 | with | with | with | without | without | with | without | without | without | without | without |
| 34 | with | with | with | without | without | without | without | without | with | without | without |
| 35 | with | with | without | without | without | with | without | without | with | without | without |
| 36 | with | with | without | with | without | without | with | without | without | without | without |
| 37 | with | with | without | with | without | without | without | without | without | with | without |
| 38 | with | with | without | without | without | without | with | without | without | with | without |
| 39 | with | with | without | without | with | without | without | with | without | without | without |
| 40 | with | with | without | without | with | without | without | without | without | without | with |
| 41 | with | with | without | without | without | without | without | with | without | without | with |
| 42 | with | with | with | without | without | with | without | without | with | without | without |
| 43 | with | with | without | with | without | without | with | without | without | with | without |
| 44 | with | with | without | without | with | without | without | with | without | without | with |

**Supplementary Table 4. Univariate analysis of patient characteristics for overall survival (OS) and disease-free survival (DFS) in patients with colorectal cancer (CRC)**

|  |  |  | **OS** | | |  | **DFS** | | |
| --- | --- | --- | --- | --- | --- | --- | --- | --- | --- |
| **Characteristic** |  | **n** | **Events (%)** | **HR (95% CI)** | ***P* value** |  | **Events (%)** | **HR (95% CI)** | ***P* value** |
| Sex | male | 106 | 31 (29) | 1 | .59 |  | 43 (41) | 1 | .63 |
|  | female | 82 | 21 (26) | 0.86 (0.49 - 1.5) |  |  | 30 (37) | 0.89 (0.56 - 1.42) |  |
| Age, y | ＜65y | 111 | 18 (16) | 1 | **.001** |  | 31 (28) | 1 | **.001** |
|  | ≥65y | 77 | 33 (43) | 3.11 (1.75 - 5.53) |  |  | 42 (55) | 2.18 (1.37 - 3.47) |  |
| Clinical diagnosis | colon cancer | 105 | 33 (31) | 1 | **.09** |  | 44 (42) | 1 | .29 |
|  | rectal cancer | 83 | 18 (22) | 0.61 (0.34 - 1.08) |  |  | 29 (35) | 0.78 (0.49 - 1.24) |  |
| Tumour site | right | 52 | 16 (31) | 1 | .20 |  | 22 (42) | 1 | .42 |
|  | left | 52 | 17 (33) | 0.89 (0.45 - 1.76) | .74 |  | 22 (42) | 0.94 (0.52 - 1.69) | .83 |
|  | rectum | 83 | 18(22) | 0.56 (0.29 - 1.10) | **.09** |  | 29 (35) | 0.74 (0.43 - 1.29) | .29 |
| Mucinous | no | 152 | 38 (25) | 1 | .29 |  | 56 (37) | 1 | .36 |
|  | yes | 36 | 13 (36) | 1.40 (0.75 - 2.64) |  |  | 17 (47) | 1.29 (0.75 - 2.22) |  |
| Differentiation | poor | 40 | 17 (43) | 1 | **.03** |  | 23 (58) | 1 | **.008** |
|  | moderate to well | 140 | 33 (24) | 0.52 (0.29 - 0.94) |  |  | 48 (34) | 0.51 (0.31 - 0.84) |  |
| T stage | T3 | 88 | 18 (20) | 1 | **.04** |  | 30 (34) | 1 | .21 |
|  | T4 | 100 | 33 (33) | 1.81 (1.02 - 3.22) |  |  | 43 (43) | 1.35 (0.85 - 2.15) |  |
| Lymphovascular invasion | no | 149 | 41 (28) | 1 | .72 |  | 60 (40) | 1 | .31 |
|  | yes | 39 | 10 (26) | 0.88 (0.44 - 1.76) |  |  | 13 (33) | 0.73 (0.4 - 1.33) |  |
| CEA (ng/ml) | ＜5 | 124 | 23 (19) | 1 | **.001** |  | 39 (31) | 1 | **.001** |
|  | ≥5 | 64 | 28 (44) | 2.66 (1.53 - 4.62) |  |  | 34 (53) | 2.13 (1.34 - 3.37) |  |
| Chemotherapy | no | 76 | 21 (28) | 1 | .49 |  | 27 (36) | 1 | .53 |
|  | yes | 112 | 30 (27) | 0.82 (0.47 - 1.43) |  |  | 46 (41) | 1.16 (0.72 - 1.87) |  |
| Radiotherapy | no | 163 | 42 (26) | 1 | .78 |  | 57 (35) | 1 | .71 |
|  | yes | 15 | 5 (33) | 1.14 (0.45 - 2.9) |  |  | 6 (40) | 1.17 (0.51 - 2.72) |  |

**Supplementary Table 5. Univariate analysis of tumor infiltrating lymphocytes (TILs) for overall survival (OS) and disease-free survival (DFS) in patients with colorectal cancer (CRC)**

|  |  | **OS** | |  | **DFS** | |
| --- | --- | --- | --- | --- | --- | --- |
| **Total region** | **Variable** | **HR (95% CI)** | ***P* value** |  | **HR (95% CI)** | ***P* value** |
| T cells | CD3+ | 0.97 (0.92 - 1.03) | .34 |  | 1.01 (0.98 - 1.04) | .62 |
| CD4+ helper T cells | CD4+ | 0.97 (0.93 - 1.01) | .16 |  | 0.98 (0.95 - 1.01) | .21 |
| CD8+ cytotoxic T cells | CD8+ | 0.82 (0.64 - 1.04) | .11 |  | 0.81 (0.66 - 0.99) | **.04** |
| Memory T cells | CD45RO+ | 0.98 (0.92 - 1.04) | .46 |  | 0.99 (0.94 - 1.03) | .55 |
| CD8+ memory T cells | CD8+CD45RO+ | 0.37 (0.1 - 1.4) | .14 |  | 0.37 (0.13 - 1.08) | **.07** |
| Macrophages | CD68+ | 1.05 (0.99 - 1.11) | .11 |  | 1.02 (0.97 - 1.08) | .36 |
| M1 macrophages | CD68+CD163- | 1.07 (1.01 - 1.15) | **.03** |  | 1.03 (0.97 - 1.1) | .30 |
| M2 macrophages | CD68+CD163+ | 1.01 (0.83 - 1.24) | .91 |  | 1.03 (0.87 - 1.22) | .72 |
| PD-L1+ macrophages | PD-L1+CD68+ | 0.79 (0.43 - 1.46) | .45 |  | 0.67 (0.35 - 1.29) | .24 |
| Tregs | CD4+FOXP3+ | 0.96 (0.54 - 1.67) | .87 |  | 1.07 (0.69 - 1.66) | .76 |
| CD3+PD-1+ T cells | CD3+PD-1+ | 0.91 (0.71 - 1.16) | .44 |  | 0.99 (0.85 - 1.16) | .95 |
| CD8+PD-1+ T cells | CD8+PD-1+ | 0.09 (0.01 - 1.47) | **.09** |  | 0.21 (0.03 - 1.39) | .11 |
| PD-L1+ cells | PD-L1+ | 0.98 (0.91 - 1.06) | .63 |  | 0.99 (0.93 - 1.05) | .75 |
