# Supplementary figures and images for: A Machine Learning–Based Scoring System to Identify High Immunoactivity Microsatellite Stability Tumors by Quantifying Similarity to Microsatellite Instability-High Tumors in Colorectal Cancers: Development and Quantitative Study
Source: JMIR Form Res. 2025 Oct 16;9:e66960. doi: 10.2196/66960 (PMC12530644; doi:10.2196/66960)

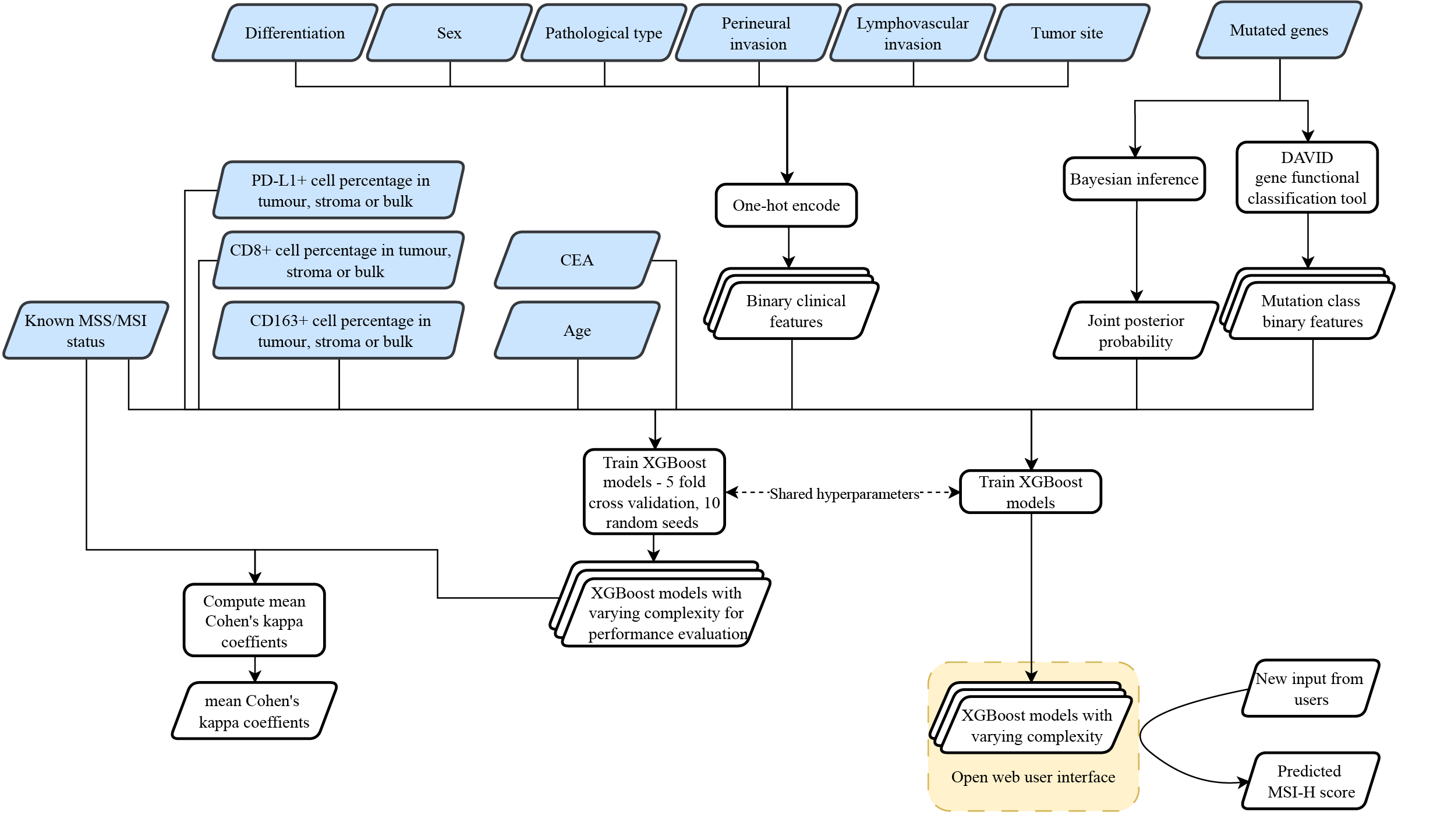

Supplement: Multimedia Appendix 2 [file formative-v9-e66960-s002.png]
