# Supplementary material for: A Machine Learning–Based Scoring System to Identify High Immunoactivity Microsatellite Stability Tumors by Quantifying Similarity to Microsatellite Instability-High Tumors in Colorectal Cancers: Development and Quantitative Study
Source: JMIR Form Res. 2025 Oct 16;9:e66960. doi: 10.2196/66960 (PMC12530644; doi:10.2196/66960)
Supplement: Multimedia Appendix 3 [file formative-v9-e66960-s003.doc]

1. Define a function called score that takes an input array as a parameter.

2. Initialize variable var0:

a. If input[1] ≥ 3.687:

i. If input[21] ≥ 1.0: var0 = 0.1693617

ii. Else:

A. If input[8] ≥ 16.725: var0 = 0.15471698

B. Else: var0 = -0.053125

b. Else:

i. If input[5] ≥ 43.0: var0 = -0.18730159

ii. Else: var0 = 0.055421688

3. Initialize variable var1:

a. If input[2] ≥ 2.504:

i. If input[2] ≥ 2.792:

A. If input[2] ≥ 6.82: var1 = 0.116018765

B. Else: var1 = -0.14021266

ii. Else: var1 = 0.1401882

b. Else:

i. If input[2] ≥ 0.0685:

A. If input[4] ≥ 1.0: var1 = -0.025534624

B. Else: var1 = -0.17311084

ii. Else: var1 = 0.08586892

4. Initialize variable var2:

a. If input[2] ≥ 1.7433231:

i. If input[2] ≥ 1.8740001:

A. If input[6] ≥ 1.0: var2 = 0.13359903

B. Else: var2 = -0.04928999

ii. Else: var2 = 0.15407658

b. Else:

i. If input[2] ≥ 0.0685:

A. If input[0] ≥ 3.5455: var2 = 0.0016782882

B. Else: var2 = -0.16685174

ii. Else: var2 = 0.04255567

5. Initialize variable var3:

a. If input[13] ≥ 0.599087: var3 = 0.13642746

b. Else:

i. If input[21] ≥ 1.0: var3 = 0.08180197

ii. Else: var3 = -0.119704306

6. Initialize variable var4:

a. If input[15] ≥ 1.0:

i. If input[8] ≥ 3.315:

A. If input[8] ≥ 91.075005: var4 = 0.13387597

B. Else: var4 = -0.14357954

ii. Else: var4 = 0.09903235

b. Else:

i. If input[8] ≥ 0.55: var4 = -0.15832432

ii. Else: var4 = 0.1130295

7. Initialize variable var5:

a. If input[1] ≥ 3.4555001: var5 = 0.11123984

b. Else:

i. If input[22] ≥ 1.0: var5 = 0.05638778

ii. Else: var5 = -0.11093491

8. Initialize variable var6:

a. If input[1] ≥ 3.3899999: var6 = 0.10016211

b. Else: var6 = -0.094145305

9. Initialize variable var7:

a. If input[13] ≥ 0.40471867: var7 = 0.07687051

b. Else:

i. If input[13] ≥ 0.029534249: var7 = -0.1493636

ii. Else: var7 = 0.05533901

10. Initialize variable var8:

a. If input[10] ≥ 1.0: var8 = -0.13358198

b. Else:

i. If input[8] ≥ 1.51: var8 = 0.047283124

ii. Else:

A. If input[8] ≥ 0.53000003: var8 = -0.13367946

B. Else: var8 = 0.066839285

11. Initialize variable var9:

a. If input[15] ≥ 1.0: var9 = 0.07934581

b. Else:

i. If input[2] ≥ 0.9075:

A. If input[2] ≥ 0.9335: var9 = -0.0099241575

B. Else: var9 = 0.17166778

ii. Else: var9 = -0.13681667

12. Initialize variable var10:

a. If input[21] ≥ 1.0:

i. If input[0] ≥ 2.186:

A. If input[0] ≥ 8.6310005: var10 = 0.11297213

B. Else: var10 = -0.112400375

ii. Else: var10 = 0.13701144

b. Else:

i. If input[0] ≥ 0.024500001:

A. If input[0] ≥ 4.8044996: var10 = 0.022901723

B. Else: var10 = -0.07944455

ii. Else: var10 = 0.08294924

13. Initialize variable var11:

a. If input[11] ≥ 1.0: var11 = -0.038614865

b. Else:

i. If input[3] ≥ 1.0:

A. If input[17] ≥ 1.0: var11 = -0.10640877

B. Else: var11 = 0.09868848

ii. Else: var11 = 0.08788528

14. Initialize variable var12:

a. If input[1] ≥ 3.379:

i. If input[1] ≥ 13.771: var12 = -0.10440486

ii. Else: var12 = 0.09245208

b. Else:

i. If input[8] ≥ 2.03: var12 = -0.12955199

ii. Else:

A. If input[8] ≥ 1.51: var12 = 0.060537186

B. Else: var12 = -0.11359862

15. Initialize variable var13:

a. If input[15] ≥ 1.0: var13 = 0.04562822

b. Else:

i. If input[0] ≥ 0.65900004: var13 = -0.08453608

ii. Else:

A. If input[0] ≥ 0.3495: var13 = 0.12474666

B. Else: var13 = -0.037533194

16. Initialize variable var14:

a. If input[13] ≥ 0.85453725: var14 = 0.09947944

b. Else:

i. If input[12] ≥ 1.0: var14 = 0.029913714

ii. Else: var14 = -0.08716019

17. Initialize variable var15:

a. If input[15] ≥ 1.0: var15 = 0.074761435

b. Else: var15 = -0.10089966

18. Initialize variable var16:

a. If input[7] ≥ 1.0: var16 = -0.12961423

b. Else:

i. If input[5] ≥ 43.0:

A. If input[5] ≥ 70.0: var16 = 0.070835955

B. Else: var16 = -0.023344042

ii. Else: var16 = 0.116462804

19. Initialize variable var17:

a. If input[5] ≥ 43.0:

i. If input[16] ≥ 1.0: var17 = 0.044099092

ii. Else: var17 = -0.061495323

b. Else: var17 = 0.09737935

20. Initialize variable var18:

a. If input[6] ≥ 1.0:

i. If input[17] ≥ 1.0: var18 = 0.099345826

ii. Else: var18 = -0.10413255

b. Else: var18 = -0.03440967

21. Initialize variable var19:

a. If input[1] ≥ 3.4555001: var19 = 0.073544465

b. Else: var19 = -0.13798614

22. Initialize variable var20:

a. If input[9] ≥ 1.0: var20 = 0.0524543

b. Else:

i. If input[6] ≥ 1.0: var20 = 0.036997914

ii. Else: var20 = -0.095599905

23. Initialize variable var21:

a. If input[16] ≥ 1.0: var21 = 0.07852443

b. Else:

i. If input[5] ≥ 76.0:

A. If input[8] ≥ 3.855: var21 = -0.09017848

B. Else: var21 = 0.14574295

ii. Else:

A. If input[5] ≥ 43.0: var21 = -0.051811058

B. Else: var21 = 0.07409403

24. Initialize variable var22:

a. If input[20] ≥ 1.0: var22 = 0.009997397

b. Else: var22 = -0.09820273

25. Initialize variable var23:

a. If input[0] ≥ 0.3735:

i. If input[0] ≥ 0.65900004: var23 = -0.04404636

ii. Else: var23 = 0.08180218

b. Else: var23 = -0.13553122

26. Initialize variable var24:

a. If input[1] ≥ 1.3975: var24 = 0.053644665

b. Else: var24 = -0.122903764

27. Initialize variable var25:

a. If input[11] ≥ 1.0: var25 = -0.091383405

b. Else:

i. If input[8] ≥ 4.21:

A. If input[8] ≥ 10.91: var25 = 0.044296842

B. Else: var25 = -0.12518792

ii. Else: var25 = 0.072844274

28. Initialize variable var26:

a. If input[5] ≥ 71.0: var26 = 0.058023136

b. Else:

i. If input[5] ≥ 60.0: var26 = -0.1168803

ii. Else: var26 = 0.016749112

29. Initialize variable var27:

a. If input[20] ≥ 1.0: var27 = 0.03980778

b. Else:

i. If input[8] ≥ 1.63: var27 = -0.12795505

ii. Else: var27 = 0.045577154

30. Initialize variable var28:

a. If input[22] ≥ 1.0: var28 = 0.048953034

b. Else: var28 = -0.044056013

31. Initialize variable var29:

a. If input[8] ≥ 11.055: var29 = 0.074248545

b. Else: var29 = -0.012567127

32. Initialize variable var30:

a. If input[13] ≥ 0.25208125: var30 = 0.068074115

b. Else:

i. If input[13] ≥ 0.03705789: var30 = -0.121024035

ii. Else:

A. If input[5] ≥ 70.0: var30 = 0.096220315

B. Else: var30 = -0.09369063

33. Initialize variable var31:

a. If input[22] ≥ 1.0: var31 = 0.047226205

b. Else:

i. If input[2] ≥ 1.6923232: var31 = 0.035660844

ii. Else: var31 = -0.07305142

34. Initialize variable var32:

a. If input[13] ≥ 0.79338396: var32 = 0.05969445

b. Else:

i. If input[2] ≥ 0.0095:

A. If input[2] ≥ 1.7865: var32 = 0.030274106

B. Else: var32 = -0.13113531

ii. Else: var32 = 0.115821674

35. Initialize variable var33:

a. If input[0] ≥ 0.34600002:

i. If input[0] ≥ 0.3565:

A. If input[13] ≥ 0.99604255: var33 = 0.059499502

B. Else: var33 = -0.06703656

ii. Else: var33 = 0.14513215

b. Else: var33 = -0.13406043

36. Initialize variable var34:

a. If input[11] ≥ 1.0: var34 = -0.06421339

b. Else: var34 = 0.06561773

37. Initialize variable var35:

a. If input[1] ≥ 3.4565:

i. If input[6] ≥ 1.0: var35 = 0.095790096

ii. Else:

A. If input[1] ≥ 5.442: var35 = 0.04075181

B. Else: var35 = -0.10104845

b. Else: var35 = -0.13233584

38. Initialize variable var36:

a. If input[9] ≥ 1.0:

i. If input[1] ≥ 1.9765: var36 = 0.10506363

ii. Else: var36 = -0.085550226

b. Else:

i. If input[15] ≥ 1.0: var36 = 0.015564539

ii. Else: var36 = -0.110470705

39. Initialize variable var37:

a. If input[0] ≥ 1.1585:

i. If input[0] ≥ 5.7215004: var37 = 0.009104463

ii. Else: var37 = -0.13203472

b. Else:

i. If input[0] ≥ 0.3495: var37 = 0.067191824

ii. Else:

A. If input[0] ≥ 0.060000002: var37 = -0.1154042

B. Else: var37 = 0.07431662

40. Initialize variable var38:

a. If input[1] ≥ 1.3975:

i. If input[2] ≥ 1.2625: var38 = 0.05560089

ii. Else:

A. If input[2] ≥ 0.042000003: var38 = -0.081688195

B. Else: var38 = 0.07232217

b. Else: var38 = -0.066007294

41. Initialize variable var39:

a. If input[9] ≥ 1.0: var39 = 0.043093517

b. Else:

i. If input[22] ≥ 1.0: var39 = 0.050066005

ii. Else: var39 = -0.118884146

42. Initialize variable var40:

a. If input[12] ≥ 1.0: var40 = 0.04956752

b. Else: var40 = -0.057384353

43. Initialize variable var41:

a. If input[12] ≥ 1.0: var41 = 0.055677123

b. Else: var41 = -0.0495016

44. Initialize variable var42:

a. If input[18] ≥ 1.0: var42 = 0.056236457

b. Else: var42 = -0.036709588

45. Initialize variable var43:

a. If input[2] ≥ 2.7725: var43 = -0.053830367

b. Else:

i. If input[2] ≥ 2.734: var43 = 0.13612321

ii. Else:

A. If input[2] ≥ 0.927: var43 = -0.044458304

B. Else: var43 = 0.059872665

46. Initialize variable var44:

a. If input[1] ≥ 1.3975: var44 = 0.040371984

b. Else: var44 = -0.1157941

47. Initialize variable var45:

a. If input[5] ≥ 43.0:

i. If input[5] ≥ 70.0: var45 = 0.060632635

ii. Else:

A. If input[2] ≥ 0.0065: var45 = -0.045125272

B. Else: var45 = 0.09916341

b. Else: var45 = 0.10046452

48. Initialize variable var46:

a. If input[8] ≥ 2.11:

i. If input[8] ≥ 6.89: var46 = 0.030241454

ii. Else: var46 = -0.13187005

b. Else:

i. If input[8] ≥ 1.51: var46 = 0.095274866

ii. Else: var46 = -0.032764085

49. Initialize variable var47:

a. If input[21] ≥ 1.0: var47 = 0.0661034

b. Else: var47 = -0.039088443

50. Initialize variable var48:

a. If input[13] ≥ 0.91505766: var48 = 0.0652815

b. Else:

i. If input[13] ≥ 0.41603118: var48 = -0.1416127

ii. Else: var48 = 0.024373677

51. Initialize variable var49:

a. If input[13] ≥ 0.79338396:

i. If input[1] ≥ 1.1485: var49 = 0.08997786

ii. Else: var49 = -0.10440864

b. Else:

i. If input[13] ≥ 0.03705789: var49 = -0.1383076

ii. Else: var49 = 0.05548625

52. Initialize variable var50:

a. If input[15] ≥ 1.0:

i. If input[11] ≥ 1.0: var50 = -0.10755433

ii. Else: var50 = 0.040241517

b. Else: var50 = -0.06280109

53. Initialize variable var51:

a. If input[0] ≥ 5.1695: var51 = 0.05437016

b. Else: var51 = -0.05764727

54. Initialize variable var52:

a. If input[5] ≥ 72.0: var52 = -0.05043145

b. Else:

i. If input[5] ≥ 71.0: var52 = 0.10822213

ii. Else:

A. If input[5] ≥ 60.0: var52 = -0.10087277

B. Else: var52 = 0.0535144

55. Initialize variable var53:

a. If input[12] ≥ 1.0: var53 = 0.061998308

b. Else: var53 = -0.05352365

56. Initialize variable var54:

a. If input[0] ≥ 0.4105: var54 = 0.037347034

b. Else: var54 = -0.11373263

57. Initialize variable var55:

a. If input[2] ≥ 0.917:

i. If input[2] ≥ 2.734: var55 = 0.060944553

ii. Else: var55 = -0.11723333

b. Else: var55 = 0.044265155

58. Initialize variable var56:

a. If input[1] ≥ 1.3975: var56 = 0.036996044

b. Else: var56 = -0.11185489

59. Initialize variable var57:

a. If input[5] ≥ 72.0:

i. If input[17] ≥ 1.0: var57 = -0.09780603

ii. Else: var57 = 0.086773194

b. Else:

i. If input[5] ≥ 52.0: var57 = 0.06258296

ii. Else:

A. If input[5] ≥ 43.0: var57 = -0.13569967

B. Else: var57 = 0.04408572

60. Initialize variable var58:

a. If input[0] ≥ 0.3495: var58 = 0.053377997

b. Else: var58 = -0.053304352

61. Initialize variable var59:

a. If input[20] ≥ 1.0: var59 = 0.03277142

b. Else: var59 = -0.0830816

62. Initialize variable var60:

a. If input[8] ≥ 2.11: var60 = -0.049539372

b. Else: var60 = 0.049073514

63. Initialize variable var61:

a. If input[2] ≥ 0.917:

i. If input[5] ≥ 72.0: var61 = -0.11796337

ii. Else:

A. If input[5] ≥ 71.0: var61 = 0.10831553

B. Else: var61 = -0.07494542

b. Else: var61 = 0.050713528

64. Initialize variable var62:

a. If input[1] ≥ 1.3975: var62 = 0.039552297

b. Else: var62 = -0.112402976

65. Initialize variable var63:

a. If input[13] ≥ 0.79338396: var63 = 0.059842207

b. Else:

i. If input[13] ≥ 0.062266424: var63 = -0.084815755

ii. Else: var63 = 0.058809716

66. Initialize variable var64:

a. If input[3] ≥ 1.0: var64 = -0.1205618

b. Else: var64 = 0.04569696

67. Initialize variable var65:

a. If input[2] ≥ 0.9075: var65 = 0.01877

b. Else: var65 = -0.09435996

68. Initialize variable var66:

a. If input[1] ≥ 4.354: var66 = 0.055874854

b. Else: var66 = -0.046661407

69. Initialize variable var67:

a. If input[12] ≥ 1.0: var67 = 0.06105993

b. Else: var67 = -0.06447073

70. Calculate weighted sum = var0 + var1 + var2 + var3 + var4 + var5 + var6 + var7 + var8 + var9 + var10 +

var11 + var12 + var13 + var14 + var15 + var16 + var17 + var18 + var19 + var20 + var21 + var22 +

var23 + var24 + var25 + var26 + var27 + var28 + var29 + var30 + var31 + var32 + var33 + var34 +

var35 + var36 + var37 + var38 + var39 + var40 + var41 + var42 + var43 + var44 + var45 + var46 +

var47 + var48 + var49 + var50 + var51 + var52 + var53 + var54 + var55 + var56 + var57 + var58 +

var59 + var60 + var61 + var62 + var63 + var64 + var65 + var66 + var67 + 0.2607461871

71. Apply sigmoid function to weighted sum to get var68:

a. If weighted sum < 0.0:

i. Set z = exp(weighted sum)

ii. var68 = z / (1.0 + z)

b. Else: var68 = 1.0 / (1.0 + exp(-weighted sum))

72. Return array containing [1.0 - var68, var68]
