# Supplementary material for: A Machine Learning–Based Scoring System to Identify High Immunoactivity Microsatellite Stability Tumors by Quantifying Similarity to Microsatellite Instability-High Tumors in Colorectal Cancers: Development and Quantitative Study
Source: JMIR Form Res. 2025 Oct 16;9:e66960. doi: 10.2196/66960 (PMC12530644; doi:10.2196/66960)
Supplement: Multimedia Appendix 4 [file formative-v9-e66960-s004.pdf]

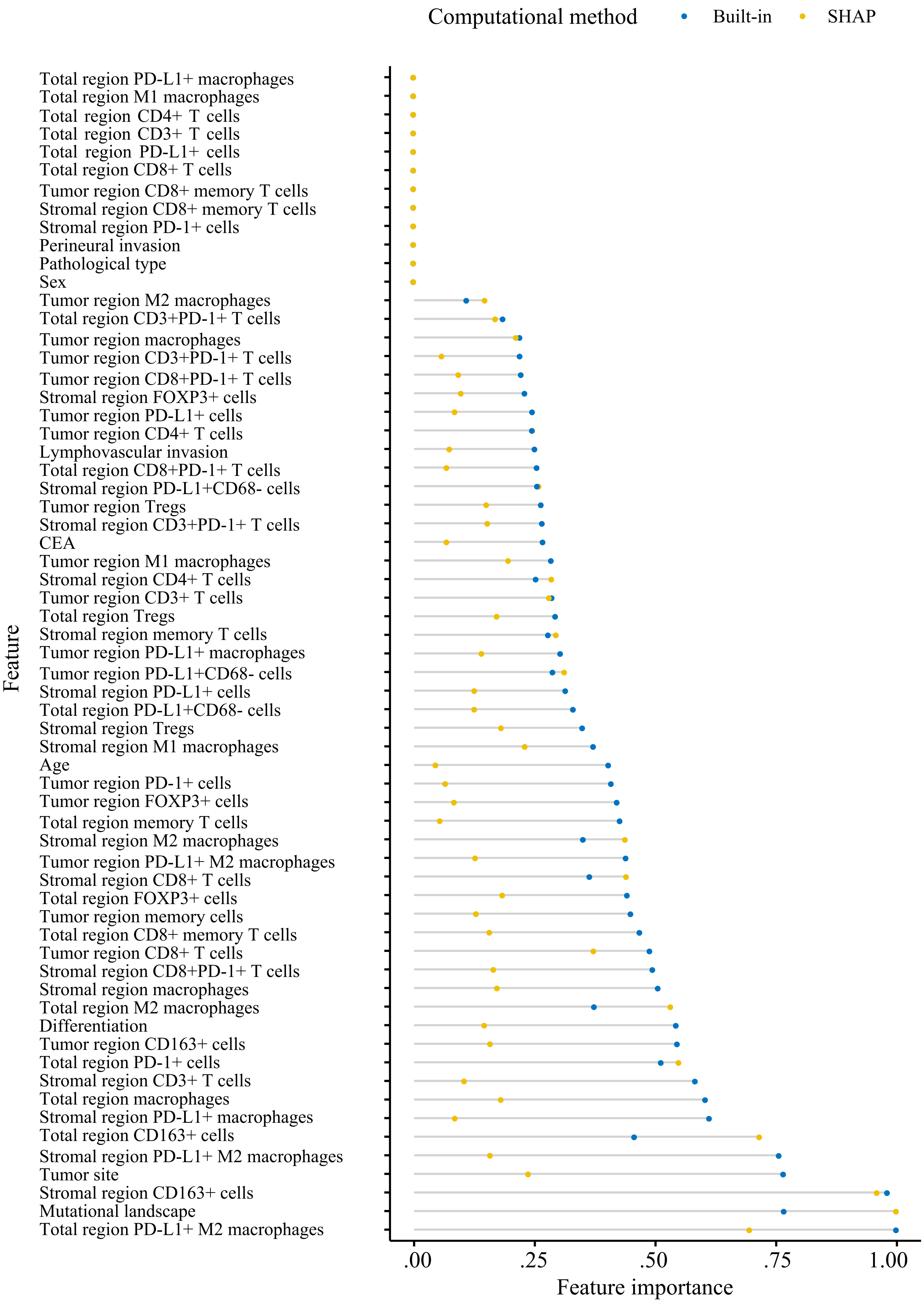

Feature importance in a large predictive model.
The feature importance ranges from .00 (minimum) to 1.00 (maximum), and each was estimated by two methods, built-in (green dot) and SHAP (yellow dot).
