# Supplementary material for: A Machine Learning–Based Scoring System to Identify High Immunoactivity Microsatellite Stability Tumors by Quantifying Similarity to Microsatellite Instability-High Tumors in Colorectal Cancers: Development and Quantitative Study
Source: JMIR Form Res. 2025 Oct 16;9:e66960. doi: 10.2196/66960 (PMC12530644; doi:10.2196/66960)
Supplement: Multimedia Appendix 5 [file formative-v9-e66960-s005.pdf]

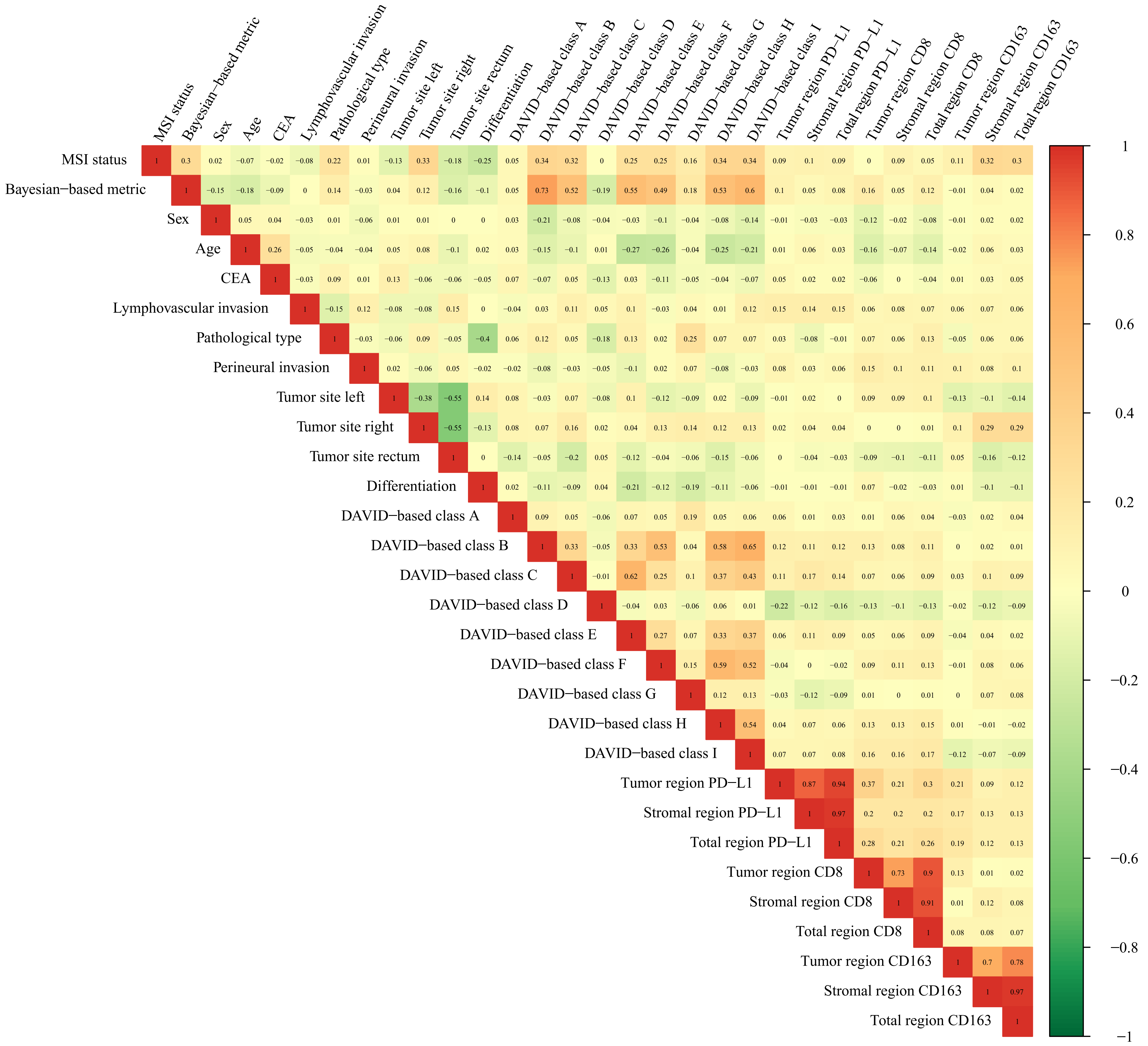

Heat map of Spearman correlation coefficients between all pairs of variables (features and targets)

The features and targets is tested by Spearman correlations analysis. The value of r ranges between 1 (dark red) and -1 (dark green) as explained in the legend corresponding to positive or negative correlations between the markers.
